# Supplementary material for: A New Model of Chronic Mycobacterium abscessus Lung Infection in Immunocompetent Mice
Source: Int J Mol Sci. 2020 Sep 9;21(18):6590. doi: 10.3390/ijms21186590 (PMC7554715; doi:10.3390/ijms21186590)
Supplement: Supplementary file 1 [file ijms-21-06590-s001.zip › Supplementary Material.docx]

**SUPPLEMENTARY MATERIAL**

**Figure S1. Time course of MA subsp. *abscessus* chronic lung infection.** CFUs in total lung and spleen. Dots represent CFUs in individual mice (number of mice = 6–11). The data were pooled from two independent experiments. Linear mixed model followed by post hoc analysis was performed (**Table S2**). Significance was calculated for each time point comparing total CFU lung and total CFU spleen. The statistic comparison of total CFU lung among the different time point are in **Table S2**.

**
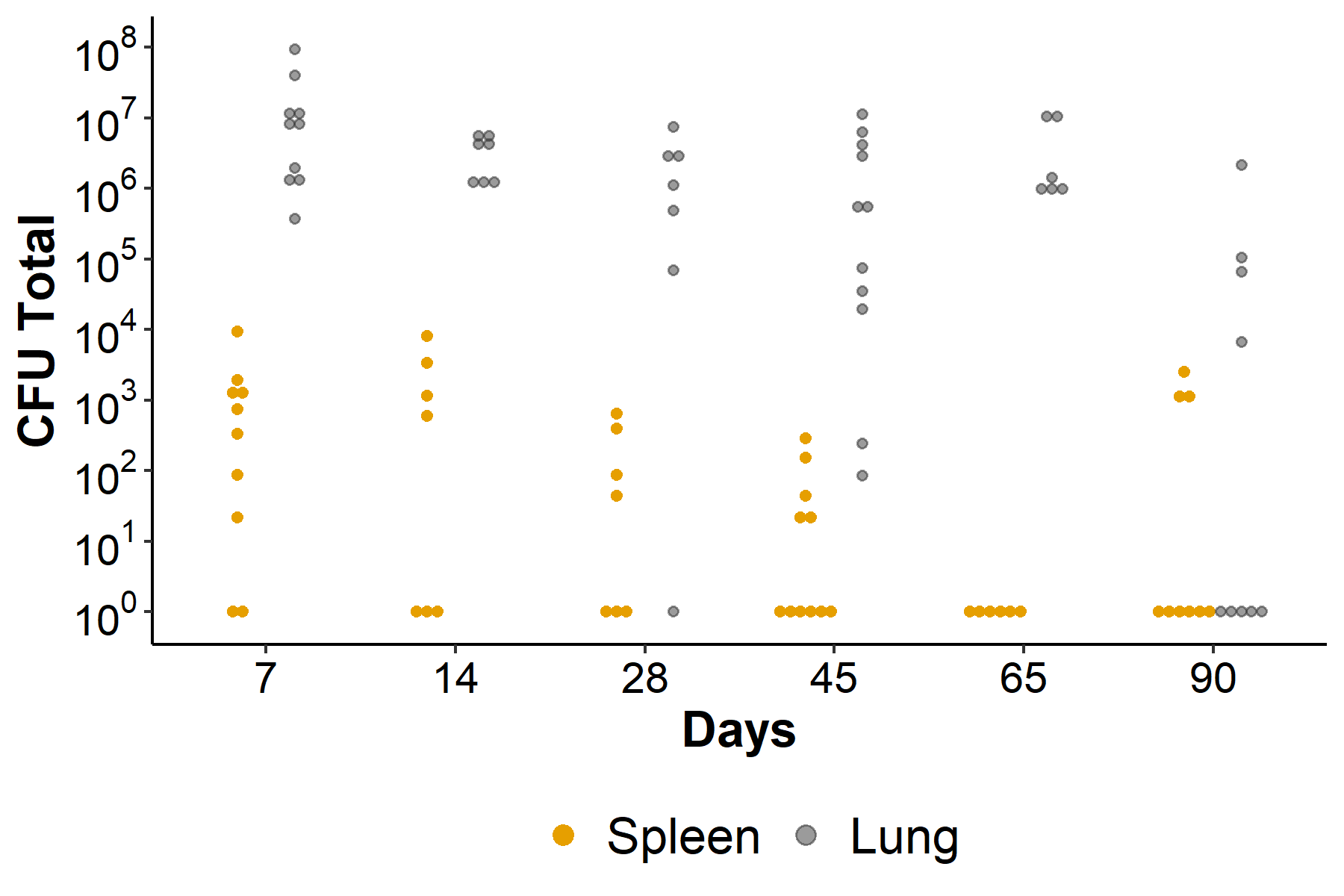
**

**Figure S2. Time course of MA subsp. *bolletii* chronic lung infection*.*** C57BL/6NCrl mice were intratracheally infected with 1x10^5^ of MA *bolletii* reference strain (ATCC 8156) embedded in agar-beads. After 7, 14, 28, 65, 90 days from infection mice were sacrificed. (**A**) Rate of chronicity (number of mice = 5–9). The data were pooled from two independent experiments (**Table S3**). (**B**) Mice were considered infected with CFUs >0 in total lung . Dots represent CFUs in individual mice. The red line represents the median values. Kruskal-Wallis test with post-hoc multiple comparisons was performed (among the time points 7, 14, 28, 45 and 90) . *p<0.05, **p<0.01, ***p<0.001 ****p<0.0001,****p<0.0001. (**C**) CFUs in total lung and spleen. Linear mixed model followed by post hoc analysis was performed (**Table S4**). Significance was calculated for each time point comparing total CFU lung and total CFU spleen.

**
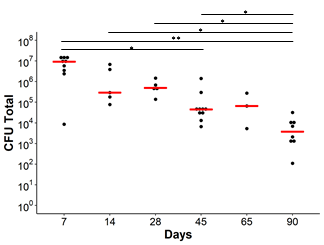
** A B C

**
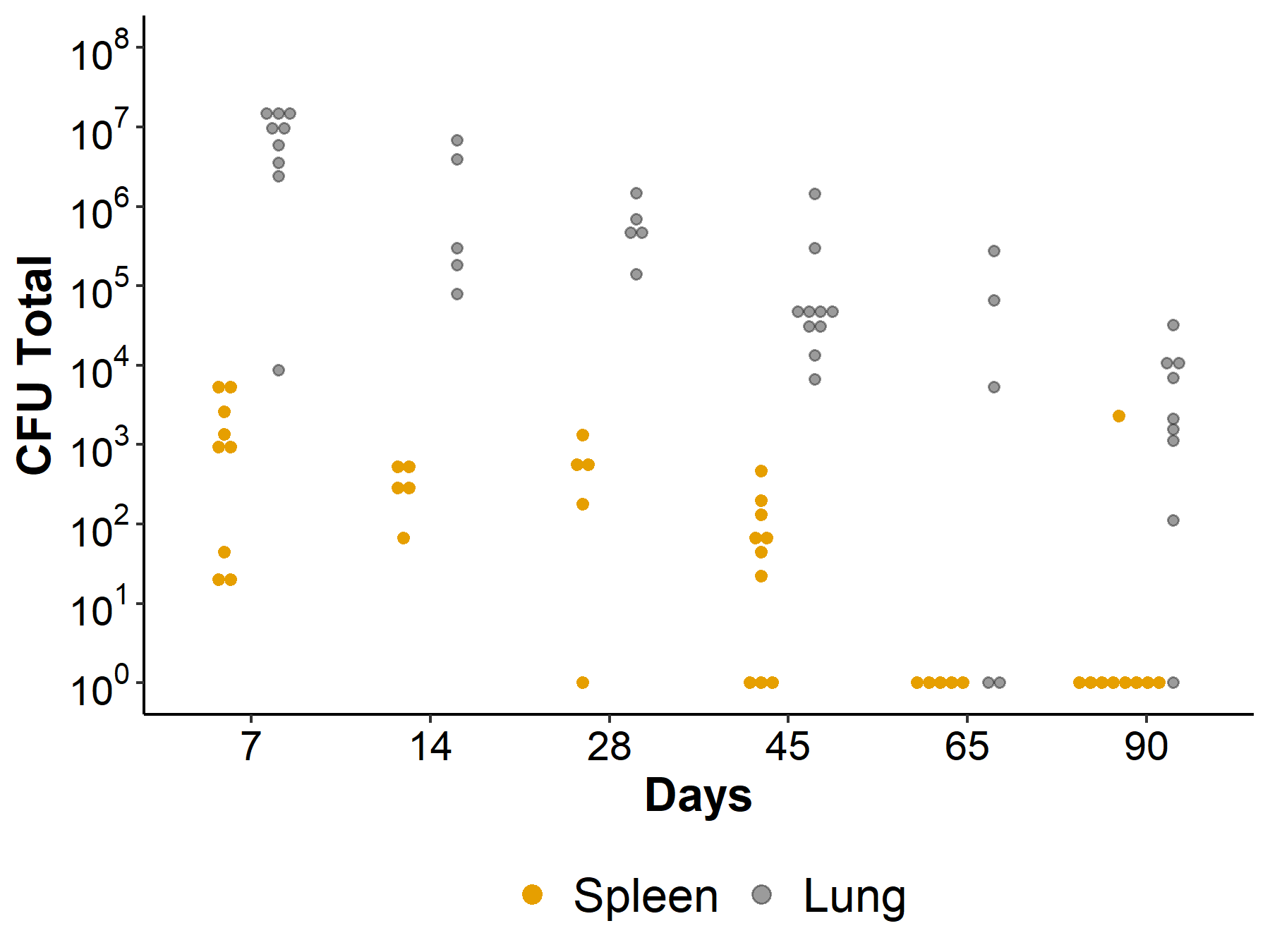

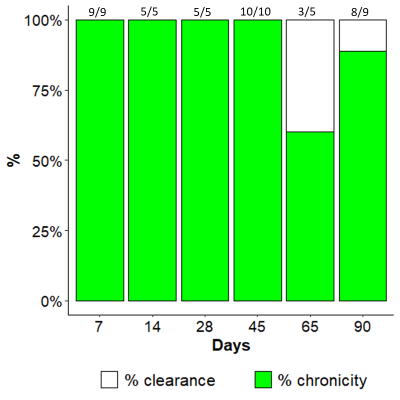
**

**Figure S3. Time course of MA subsp. *massiliense* chronic lung infection*.*** C57BL/6NCrl mice were intratracheally infected with 1x10^5^ of MA *massiliense* reference strain (ATCC 48898) embedded in agar-beads. After 7, 14, 28, 65, 90 and 180 days from infection mice were sacrificed. (**A**) Rate of chronicity (number of mice = 5–15). The data were pooled from two independent experiments (**Table S5**). (**B**) Mice were considered infected with CFUs >0 in total lung. Dots represent CFUs in individual mice. The red line represents the median values. Kruskal-Wallis test with post-hoc multiple comparisons was performed (among the time points 7, 14, 28 and 45). *p<0.05, **p<0.01, ***p<0.001 ****p<0.0001,****p<0.0001. (**C**) CFUs in total lung and spleen. Linear mixed model followed by post hoc analysis was performed (**Table S6**). Significance was calculated for each time point comparing total CFU lung and total CFU spleen.


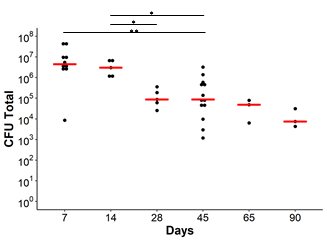
A B C


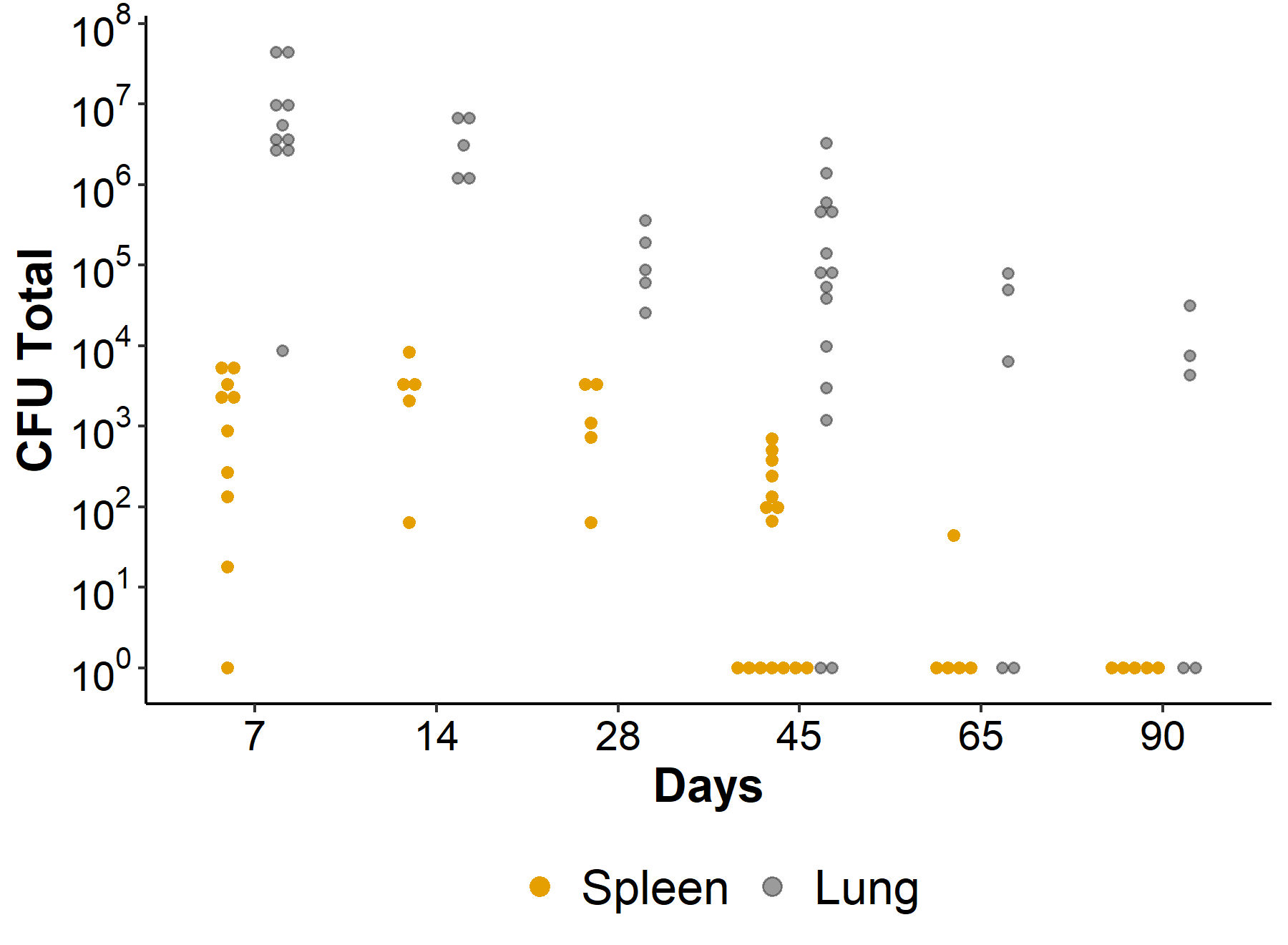

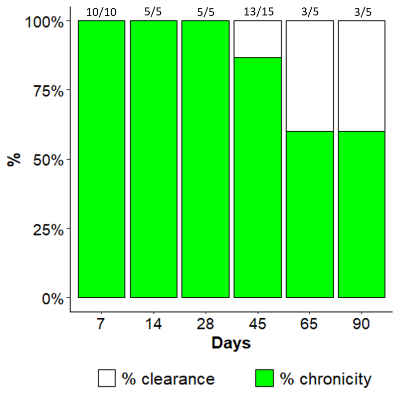


**Figure S4**. Estimated model for the body weight (**Figure S3 A**, **Table S7**) and Kaplan–Meier estimates for overall survival (days) (**Figure S3 B, Table S8**) of mice.

**
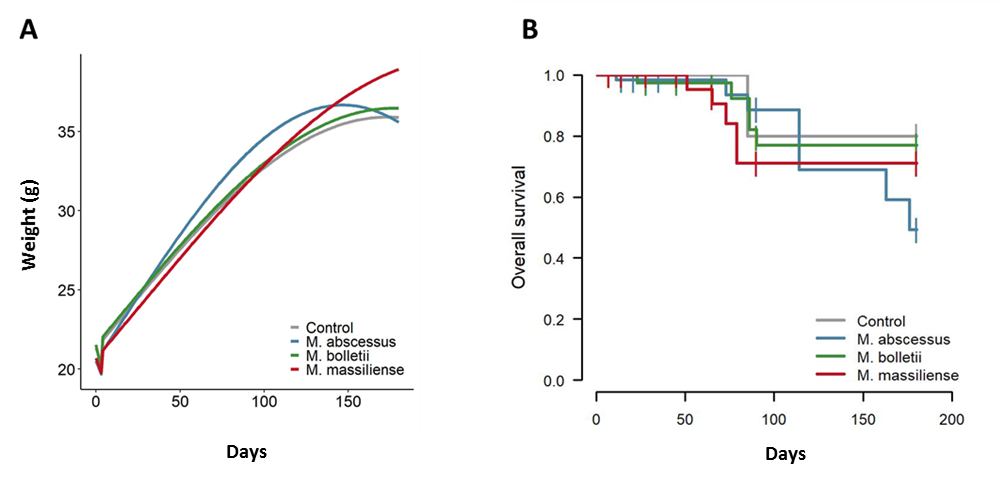
**

**Figure S5. (A)** The panel shows H&E, anti-IBA 1, anti-CD3 and anti-B220 (20X, AxioCam HRc Zeiss) stained sections of lungs after 7, 45 and 90 days from MA subsp. *abscessus* infection; scale bar: 50 μm. (**B**) H&E (**a** 5X and **b** 20X, AxioCam HRc Zeiss) and Ziehl Neelsen (**c** 20X, AxioCam HRc Zeiss) stained sections of lungs after 45 days of MA subsp *abscessus* infection; scale bar 200 μm (5X) and 50 μm (20X). Asterisk indicate beads in the bronchial lumen of a main bronchus.


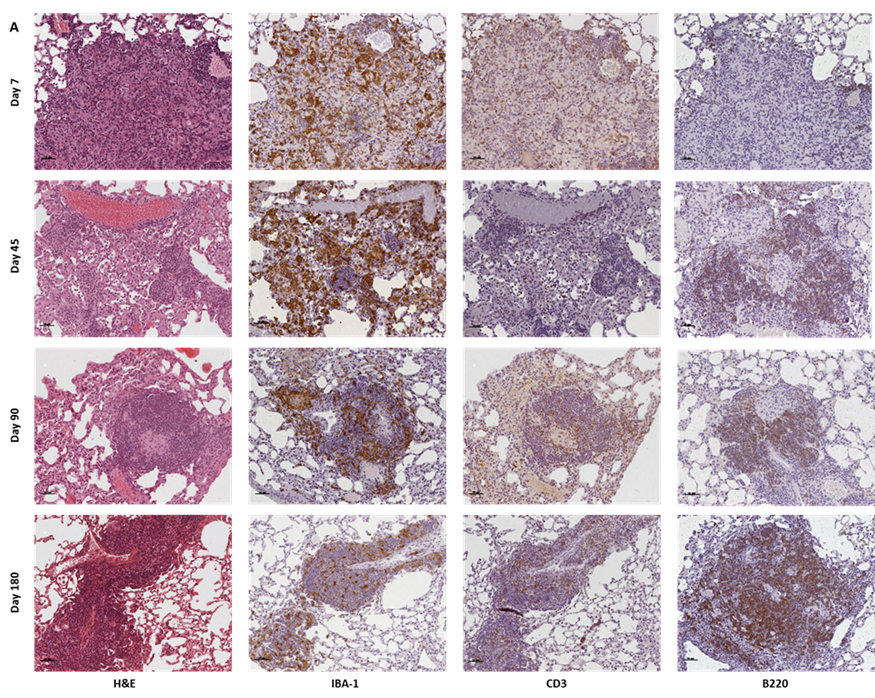


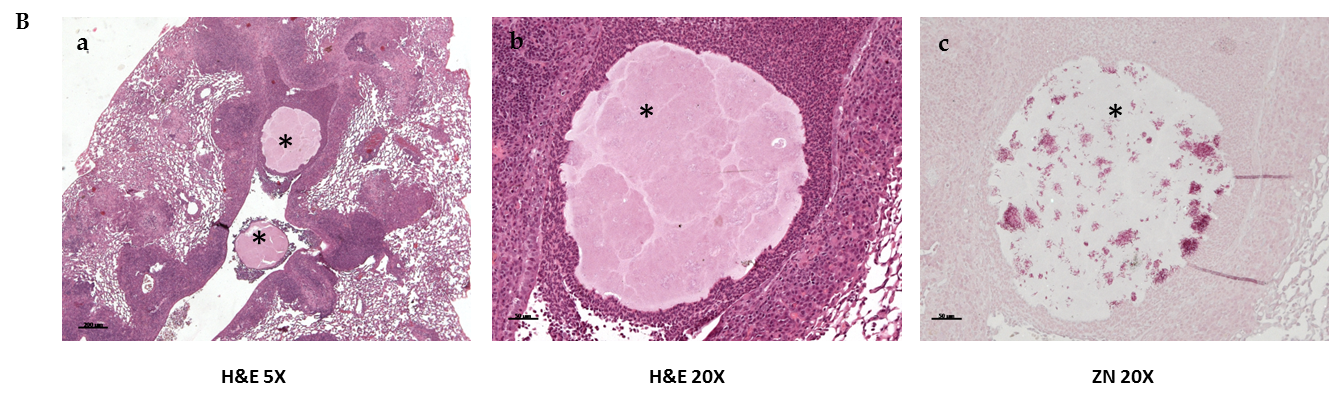


**Figure S6. Lung lesions after 7, 45 and 90 days of chronic infection with MA subsp. *bolletii* (S6A*)* and MA subsp*. massiliense* (S6B)*.*** Dots represents the lobes analyzed by Image J program with WEKA algorithm: 5 lobe images of 5 mice for each time point (25 dots for each time point). Total lung lesions were estimated as the percentage of lesioned area to total lung area in mice infected or not by MA subsp. *bolletii* and *massiliense* (line at median). Linear mixed model followed by post hoc analysis was performed (**Table S10 and S11**). Significance was calculated for each time point comparing MA subsp. *bolletii* and control and Ma subsp. *massiliense* and control *p<0.05, **p<0.01, ***p<0.001 ****p<0.0001.

**A B**

**
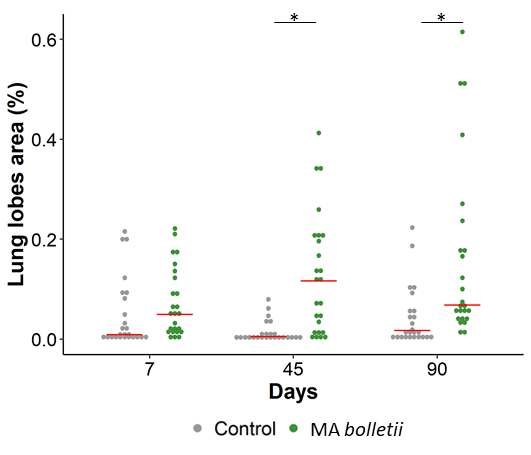

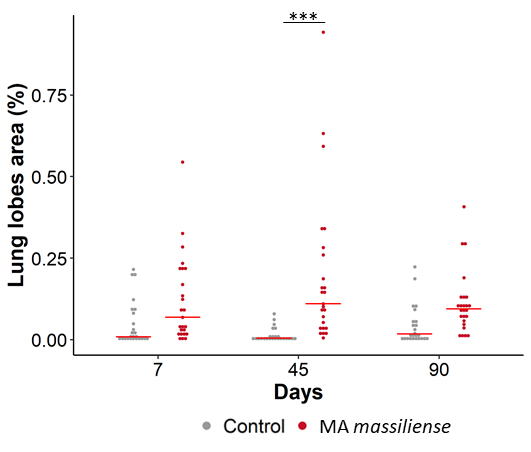
**

**Figure S7.** Cytokines/chemokines recruitment during MA subsp. *bolletii* infection. (A) IFN-γ, (B) TNF-α measured by Mouse Milliplex, were quantified in total lung of mice. At 7 and 45 days dots represent cells in individual mice selected from the group of infected mice with MA subsp. *bolletii*. The data were pooled from two independent experiments. For MA subsp. *bolletii*, statistical time effect was evaluated with Mann-Whitney Test. Statistical comparison between MA subsp. *Bolletii* and control mice at Day 7 was calculated with Mann-Whitney Test. *p<0.05, **p<0.01, ***p<0.001 ****p<0.0001.


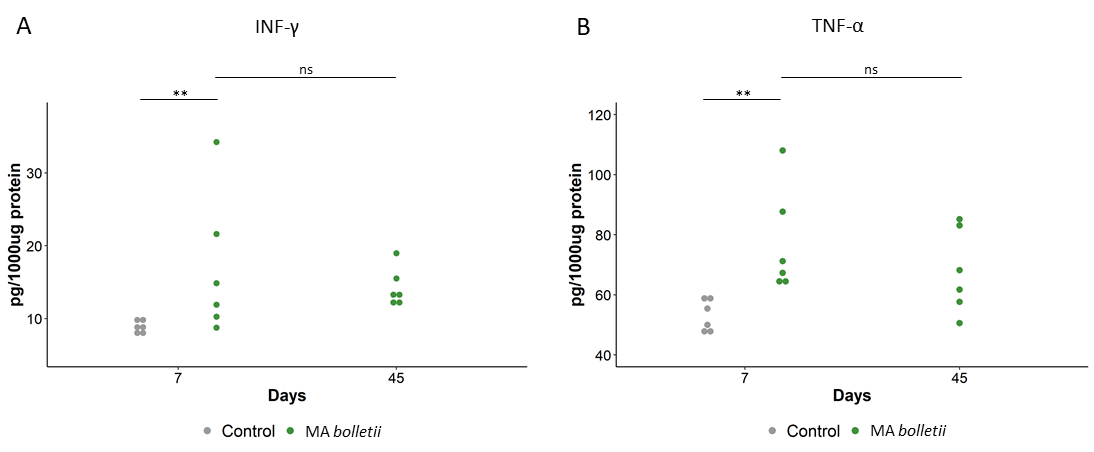


**Figure S8.** Cytokines/chemokines recruitment during MA subsp. *massiliense* infection. (A) IFN-γ, (B) TNF-α. measured by Mouse Milliplex, were quantified in total lung of mice. At 7 and 45 days dots represent cells in individual mice selected from the group of infected mice with MA subsp. *massiliense*. The data were pooled from two independent experiments. For MA subsp. *massiliense*, statistical time effect was evaluated Mann-Whitney Test. Statistical comparison between MA subsp. *massiliense* and control mice at Day 7 was calculated with Mann-Whitney Test. *p<0.05, **p<0.01, ***p<0.001 ****p<0.0001.

**
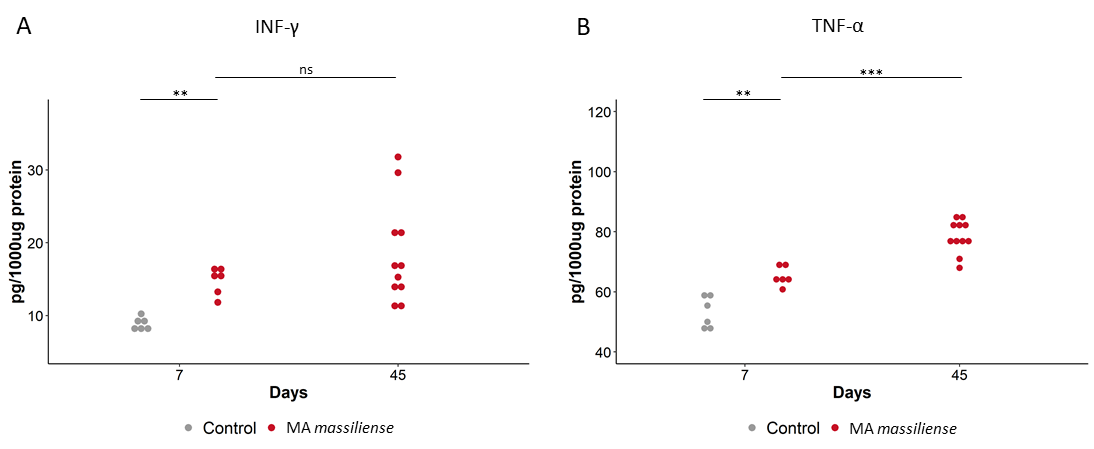
**
