# Supplementary material for: A New Model of Chronic Mycobacterium abscessus Lung Infection in Immunocompetent Mice
Source: Int J Mol Sci. 2020 Sep 9;21(18):6590. doi: 10.3390/ijms21186590 (PMC7554715; doi:10.3390/ijms21186590)
Supplement: Supplementary file 1 [file ijms-21-06590-s001.zip › Supplementary Statistical Analyses.docx]

**SUPPLEMENTARY STATISTICAL ANALYSES**

**Table S1**. Logistic regression model for chronicity (MA subsp. *abscessus,* Days 7-90).

| **Parameter** | **Estimate** | **SE** | **p-value** |
| --- | --- | --- | --- |
| Intercept | 5.8729 | 1.8978 | 0.0020 |
| Days | -0.0644 | 0.0244 | 0.0084 |

**Table S2**. Linear mixed model for MA subsp. *abscessus* total CFU lung and total CFU spleen (OrderNorm transformation). For testing differences between total CFU lung and total CFU spleen at each time point, a post-hoc analysis was performed.

| **Parameter** | **Estimate** | **SE** | **p-value** |
| --- | --- | --- | --- |
| Intercept | -0.1425 | 0.1542 | 0.3606 |
| Type (Ref=spleen) |  |  |  |
| Lung | 1.3843 | 0.1874 | **<0.0001** |
| Days (Ref=7) |  |  |  |
| Days14 | -0.1473 | 0.2403 | 0.543 |
| Days28 | -0.2908 | 0.2403 | 0.2327 |
| Days45 | -0.4318 | 0.2131 | 0.0488 |
| **Days65** | **-0.7447** | **0.2518** | **0.005** |
| Days90 | -0.4002 | 0.2241 | 0.081 |
| typeLung:Days14 | -0.122 | 0.2862 | 0.6721 |
| typeLung:Days28 | -0.1603 | 0.295 | 0.5898 |
| typeLung:Days45 | -0.1759 | 0.2548 | 0.4937 |
| typeLung:Days65 | 0.452 | 0.2995 | 0.1387 |
| **typeLung:Days90** | -1.113 | 0.2674 | **0.0004** |

| **Comparison** | **Days** | **Estimate** | **SE** | **p.value** |
| --- | --- | --- | --- | --- |
| Spleen – Lung | **7** | **-1.3843** | **0.1874** | **<0.0001** |
| Spleen – Lung | **14** | **-1.2623** | **0.2163** | **<0.0001** |
| Spleen – Lung | **28** | **-1.224** | **0.2278** | **<0.0001** |
| Spleen – Lung | **45** | **-1.2084** | **0.1725** | **<0.0001** |
| Spleen – Lung | **65** | **-1.8363** | **0.2336** | **<0.0001** |
| Spleen – Lung | 90 | -0.2713 | 0.1908 | 0.1623 |

**Table S3**. Logistic regression model for chronicity (MA subsp. *bolletii*, Days 7-90).

| **Parameter** | **Estimate** | **SE** | **p-value** |
| --- | --- | --- | --- |
| Intercept | 4.8601 | 1.8843 | 0.0099 |
| Days | -0.0390 | 0.0249 | 0.1177 |

**Table S4**. Linear mixed model for MA *bolletii* total CFU lung and total CFU spleen (OrderNorm transformation). For testing differences between total CFU lung and total CFU spleen at each time point, a post-hoc analysis was performed.

| **Parameter** | **Estimate** | **SE** | **p-value** |
| --- | --- | --- | --- |
| Intercept | -0.0457 | 0.146 | 0.7558 |
| Type (Ref=spleen) |  |  |  |
| Lung | 1.6838 | 0.2065 | **<0.0001** |
| Days (Ref=7) |  |  |  |
| Days14 | -0.1275 | 0.2443 | 0.6049 |
| Days28 | -0.2252 | 0.2443 | 0.3626 |
| **Days45** | **-0.4915** | **0.2013** | **0.0195** |
| **Days65** | **-0.9995** | **0.2443** | **0.0002** |
| **Days90** | **-0.859** | **0.2065** | **0.0002** |
| typeLung:Days14 | -0.3837 | 0.3455 | 0.274 |
| typeLung:Days28 | -0.3406 | 0.3455 | 0.3306 |
| typeLung:Days45 | -0.4692 | 0.2846 | 0.1077 |
| typeLung:Days65 | -0.6647 | 0.3455 | 0.0621 |
| **typeLung:Days90** | **-0.6808** | **0.292** | **0.0253** |

| **Comparison** | **Days** | **estimate** | **SE** | **p.value** |
| --- | --- | --- | --- | --- |
| Spleen – Lung | 7 | -1.6838 | 0.2065 | **<0.0001** |
| Spleen – Lung | 14 | -1.3001 | 0.277 | **<0.0001** |
| Spleen – Lung | 28 | -1.3431 | 0.277 | **<0.0001** |
| Spleen – Lung | 45 | -1.2146 | 0.1959 | **<0.0001** |
| Spleen – Lung | 65 | -1.019 | 0.277 | **0.0007** |
| Spleen – Lung | 90 | -1.003 | 0.2065 | **<0.0001** |

**Table S5**. Logistic regression model for chronicity (MA subsp. *massiliense*, Days 7-90).

|  | **Estimate** | **SE** | **p-value** |
| --- | --- | --- | --- |
| Intercept | 4.4041 | 1.3127 | 0.0008 |
| Days | -0.0504 | 0.0203 | 0.0133 |

**Table S6**. Linear mixed model for MA subsp. *massiliense* total CFU lung and total CFU spleen (OrderNorm transformation). For testing differences between total CFU lung and total CFU spleen at each time point, a post-hoc analysis was performed.

| **Parameter** | | **Estimate** | | **SE** | | **p-value** | |  |
| --- | --- | --- | --- | --- | --- | --- | --- | --- |
| Intercept | | -0.0873 | | 0.1589 | | 0.5859 | |  |
| Type (Ref=spleen) | |  | |  | |  | |  |
| Lung | | 1.6572 | | 0.2089 | | **<0.0001** | |  |
| Days (Ref=7) | |  | |  | |  | |  |
| Days14 | | 0.1792 | | 0.2752 | | 0.5189 | |  |
| Days28 | | 0.074 | | 0.2752 | | 0.7895 | |  |
| **Days45** | | **-0.4709** | | **0.2052** | | **0.0272** | |  |
| **Days65** | | **-0.7648** | | **0.2752** | | **0.0084** | |  |
| **Days90** | | **-0.8801** | | **0.2752** | | **0.0027** | |  |
| typeLung:Days14 | | -0.4138 | | 0.3618 | | 0.2597 | |  |
| **typeLung:Days28** | | **-0.9309** | | **0.3618** | | **0.014** | |  |
| **typeLung:Days45** | | **-0.6101** | | **0.2697** | | **0.0293** | |  |
| **typeLung:Days65** | | **-0.8664** | | **0.3618** | | **0.0215** | |  |
| **typeLung:Days90** | | **-0.8518** | | **0.3618** | | **0.0237** | |  |
| **Comparison** | **Days** | | **estimate** | | **SE** | | **p-value** | |
| Spleen – Lung | **7** | | **-1.6572** | | **0.2089** | | **<0.0001** | |
| Spleen – Lung | **14** | | **-1.2434** | | **0.2954** | | **0.0004** | |
| Spleen – Lung | **28** | | **-0.7262** | | **0.2954** | | **0.0185** | |
| Spleen – Lung | **45** | | **-1.047** | | **0.1705** | | **<0.0001** | |
| Spleen – Lung | **65** | | **-0.7908** | | **0.2954** | | **0.0108** | |
| Spleen – Lung | **90** | | **-0.8053** | | **0.2954** | | **0.0095** | |

**Table S7**. Linear mixed-effects model for Body weight (log10 transformation). Different trends were allowed for time≤3 days (post infection) and time >3 days (post infection). For time >3 days, both linear and quadratic terms for time were included in the mixed models to account for the nonlinear trajectories of body weight over time.

|  | **Estimate** | **SE** | **p-value** |
| --- | --- | --- | --- |
| Intercept | 1.3288 | 0.0053 | <0.0001 |
| Type (Ref=Control) |  |  |  |
| MA. *abscessus* | -0.0173 | 0.0057 | 0.0028 |
| MA. *bolletii* | 0.0041 | 0.0059 | 0.4879 |
| MA. *massiliense* | -0.0131 | 0.0059 | 0.0262 |
| Days^2 | 0.0000 | 0.0000 | <0.0001 |
| Days*I(Days≤3) | -0.0067 | 0.0016 | <0.0001 |
| Days*I(Days>3) | 0.0026 | 0.0001 | <0.0001 |
| MA. *abscessus*:Days^2 | 0.0000 | 0.0000 | <0.0001 |
| MA. *bolletii*:Days^2 | 0.0000 | 0.0000 | 0.6717 |
| MA. *massiliense*:Days2 | 0.0000 | 0.0000 | 0.0041 |
| I(Days≤3):Days:MA. *abscessus* | 0.0008 | 0.0017 | 0.6142 |
| I(Days>3):Days:MA. *abscessus* | 0.0008 | 0.0001 | <0.0001 |
| I(Days≤3):Days:MA. *bolletii* | -0.0046 | 0.0018 | 0.0109 |
| I(Days>3):Days:MA. *bolletii* | 0.0000 | 0.0001 | 0.7792 |
| I(Days≤3):Days:MA. *massiliense* | 0.0001 | 0.0018 | 0.9447 |
| I(Days>3):Days:MA. *massiliense* | 0.0000 | 0.0001 | 0.9379 |

**Table S8.** Mortality rate statistics.

| **7 days post infection (p.i)** | **Total**  **Number** | **Survived**  **Number (%)** | **Dead**  **Number (%)** |
| --- | --- | --- | --- |
| Control | 12 | 12(100%) | 0(0%) |
| MA. *abscessus* | 74 | 74(100%) | 0(0%) |
| MA. *bolletii* | 54 | 54(100%) | 0(0%) |
| MA. *massiliense* | 56 | 56(100%) | 0(0%) |

| **7 days p.i - 14 days p.i.** | **Total**  **Number** | **Survived**  **Number (%)** | **Dead**  **Number (%)** |
| --- | --- | --- | --- |
| Control | 8 | 8(100%) | 0(0%) |
| MA. *abscessus* | 64 | 63(98.44%) | 1(1.56%) |
| MA. *bolletii* | 45 | 45(100%) | 0(0%) |
| MA. *massiliense* | 46 | 46(100%) | 0(0%) |

| **14 days p.i. 28 days p.i.** | **Total**  **Number** | **Survived**  **Number (%)** | **Dead**  **Number (%)** |
| --- | --- | --- | --- |
| Control | 8 | 8(100%) | 0(0%) |
| MA. *abscessus* | 49 | 49(100%) | 0(0%) |
| MA. *bolletii* | 40 | 39(97.5%) | 1(2.5%) |
| MA. *massiliense* | 41 | 41(100%) | 0(0%) |

| **28 days p.i. -45 days p.i.** | **Total**  **Number** | **Survived**  **Number (%)** | **Dead**  **Number (%)** |
| --- | --- | --- | --- |
| Control | 8 | 8(100%) | 0(0%) |
| MA. *abscessus* | 34 | 34(100%) | 0(0%) |
| MA. *bolletii* | 34 | 34(100%) | 0(0%) |
| MA. *massiliense* | 36 | 36(100%) | 0(0%) |

| **45 days p.i. 65 days p.i.** | **Total**  **Number** | **Survived**  **Number (%)** | **Dead**  **Number (%)** |
| --- | --- | --- | --- |
| Control | 8 | 8(100%) | 0(0%) |
| MA. *abscessus* | 23 | 23(100%) | 0(0%) |
| MA. *bolletii* | 24 | 24(100%) | 0(0%) |
| MA. *massiliense* | 21 | 19(90.48%) | 2(9.52%) |

| **65days p.i. -90 days p.i.** | **Total**  **Number** | **Survived**  **Number (%)** | **Dead**  **Number (%)** |
| --- | --- | --- | --- |
| Control | 5 | 4(80%) | 1(20%) |
| MA. *abscessus* | 20 | 18(90%) | 2(10%) |
| MA. *bolletii* | 19 | 15(78.95%) | 4(21.05%) |
| MA. *massiliense* | 14 | 11(78.57%) | 3(21.43%) |

| **90 p.i. - 180 p.i.** | **Total**  **Number** | **Survived**  **Number (%)** | **Dead**  **Number (%)** |
| --- | --- | --- | --- |
| Control | 4 | 4(100%) | 0(0%) |
| MA. *abscessus* | 9 | 5(55.56%) | 4(44.44%) |
| MA. *bolletii* | 6 | 6(100%) | 0(0%) |
| MA. *massiliense* | 6 | 6(100%) | 0(0%) |

**Table S9.** Linear mixed model of tissue damage during MA subsp. *abscessus* chronic infection (square root transformation). Post-hoc analysis after LME was performed, comparing Control and MA *abscessus* at each time point.

| **Parameter** | | | **Estimate** | | **SE** | | **p-value** | | |  |  |
| --- | --- | --- | --- | --- | --- | --- | --- | --- | --- | --- | --- |
| Intercept | | | 0.1691 | | 0.0563 | | 0.0033 | | |  |  |
| Days (Ref=Days7) | | |  | |  | |  | | |  |  |
| Days45 | | | -0.0767 | | 0.0796 | | 0.3454 | | |  |  |
| Days90 | | | -0.0001 | | 0.0796 | | 0.9988 | | |  |  |
| Species (Ref=Control) | | |  | |  | |  | | |  |  |
| **MA abscessus** | | | **0.2318** | | **0.0796** | | **0.0077** | | |  |  |
| Days45:MA *abscessus* | | | -0.0370 | | 0.1126 | | 0.7456 | | |  |  |
| Days90:MA *abscessus* | | | -0.1155 | | 0.1126 | | 0.3152 | | |  |  |
|  |  | | | | | | |  | | |  |
| **Comparison** | | **Days** | | **estimate** | | **SE** | | | **p.value** | | |
| Control - MA. *abscessus* | | **7** | | **-0.232** | | **0.0796** | | | **0.0077** | | |
| Control - MA. *abscessus* | | **45** | | **-0.195** | | **0.0796** | | | **0.0221** | | |
| Control - MA. *abscessus* | | 90 | | -0.116 | | 0.0796 | | | 0.1572 | | |

**Table S10.** Linear mixed model of tissue damage during MA subsp. *bolletii* chronic infection (sqrt transformation). Post-hoc analysis after LME was performed, comparing Control and MA *bolletii* at each time point.

| **Parameter** | | | **Estimate** | | **SE** | | **p-value** | | |  |  |
| --- | --- | --- | --- | --- | --- | --- | --- | --- | --- | --- | --- |
| Intercept | | | 0.1691 | | 0.0546 | | 0.0024 | | |  |  |
| Days (Ref=Days7) | | |  | |  | |  | | |  |  |
| Days45 | | | -0.0767 | | 0.0772 | | 0.3309 | | |  |  |
| Days90 | | | -0.0001 | | 0.0772 | | 0.9988 | | |  |  |
| Species (Ref=Control) | | |  | |  | |  | | |  |  |
| MA bolletii | | | 0.0687 | | 0.0772 | | 0.3829 | | |  |  |
| Days45:MA *bolletii* | | | 0.1475 | | 0.1092 | | 0.1895 | | |  |  |
| Days90:MA *bolletii* | | | 0.1116 | | 0.1092 | | 0.3171 | | |  |  |
|  |  | | | | | | |  | | |  |
| **Comparison** | | **Days** | | **estimate** | | **SE** | | | **p.value** | | |
| Control - MA. *bolletii* | | 7 | | -0.0687 | | 0.0772 | | | 0.3829 | | |
| Control - MA. *bolletii* | | **45** | | **-0.2162** | | **0.0772** | | | **0.01** | | |
| Control - MA. *bolletii* | | **90** | | **-0.1803** | | **0.0772** | | | **0.0283** | | |

**Table S11.** Linear mixed model of tissue damage during MA subsp. *massiliense* chronic infection (sqrt transformation). Post-hoc analysis after LME was performed, comparing Control and MA *massiliense* at each time point.

| **Parameter** | | | **Estimate** | | **SE** | | **p-value** | | |  |  |
| --- | --- | --- | --- | --- | --- | --- | --- | --- | --- | --- | --- |
| Intercept | | | 0.1691 | | 0.0550 | | 0.0026 | | |  |  |
| Days (Ref=Days7) | | |  | |  | |  | | |  |  |
| Days45 | | | -0.0767 | | 0.0778 | | 0.3344 | | |  |  |
| Days90 | | | -0.0001 | | 0.0778 | | 0.9988 | | |  |  |
| Species (Ref=Control) | | |  | |  | |  | | |  |  |
| MA massiliense | | | 0.1276 | | 0.0778 | | 0.1141 | | |  |  |
| Days45:MA *massiliense* | | | 0.1620 | | 0.1100 | | 0.1540 | | |  |  |
| Days90:MA *massiliense* | | | 0.0100 | | 0.1100 | | 0.9286 | | |  |  |
|  |  | | | | | | |  | | |  |
| **Comparison** | | **Days** | | **estimate** | | **SE** | | | **p.value** | | |
| Control - MA. *massiliense* | | 7 | | -0.128 | | 0.0778 | | | 0.1141 | | |
| Control - MA. *massiliense* | | **45** | | **-0.29** | | **0.0778** | | | **0.0011** | | |
| Control - MA. *massiliense* | | 90 | | -0.138 | | 0.0778 | | | 0.0898 | | |
